# Incremental value of quantitative SPECT/CT integrated parameters in a multimodal machine-learning model for differentiating spinal tuberculosis from pyogenic spondylitis

**DOI:** 10.3389/fcimb.2026.1897479

**Published:** 2026-08-18

**Authors:** Jiaxing Wang, Xingyu Duan, Jiong Wang, Mengqi Zhu, Yuxin Gao, Yingqin Jia, Qian Zhao, Ningkui Niu

**Affiliations:** 1 Department of Orthopedics, General Hospital of Ningxia Medical University, Yinchuan, Ningxia, China; 2 Ningxia Medical University, Yinchuan, Ningxia, China; 3 Key Laboratory of Clinical Pathogenic Microorganisms, General Hospital of Ningxia Medical University, Yinchuan, Ningxia, China; 4 Department of Nuclear Medicine, General Hospital of Ningxia Medical University, Yinchuan, Ningxia, China; 5 Research Center for Prevention and Control of Bone and Joint Tuberculosis, General Hospital of Ningxia Medical University, Yinchuan, Ningxia, China

**Keywords:** differential diagnosis, machine learning, pyogenic spondylitis, SPECT/CT, spinal tuberculosis

## Abstract

**Objective:**

To develop and internally validate a multimodal machine-learning model integrating clinical characteristics, laboratory markers, conventional imaging findings, and quantitative SPECT/CT parameters for differentiating spinal tuberculosis (STB) from pyogenic spondylitis (PS), and to evaluate the incremental diagnostic value of quantitative SPECT/CT parameters.

**Methods:**

This retrospective study included 96 patients with STB and 69 with PS confirmed by microbiological, molecular, or histopathological examinations between September 2024 and May 2026. Demographic characteristics, laboratory markers, conventional imaging findings, and quantitative SPECT/CT parameters were collected. Two prespecified integrated quantitative SPECT/CT parameters, LBI_SUVmax_ and LBI_SUVmean_, were used to construct a baseline model, a quantitative SPECT/CT-only model, and a fusion model. Elastic Net logistic regression was used for the primary analysis, and radial basis function support vector machine (RBF-SVM) was used for robustness analysis. Internal validation was performed using repeated stratified nested cross-validation. Model performance was evaluated using the area under the receiver operating characteristic curve (AUC), Brier score, and calibration measures. Incremental diagnostic value was assessed using 2, 000 patient-level paired bootstrap replicates. Two prespecified sensitivity analyses were also performed.

**Results:**

All seven integrated quantitative SPECT/CT parameters were significantly higher in the PS group than in the STB group (all *P* < 0.01). In the Elastic Net analysis, the baseline, quantitative SPECT/CT-only, and fusion models achieved AUCs of 0.788, 0.871, and 0.899, respectively. Compared with the baseline model, the fusion model increased the AUC by 0.110 (95% CI, 0.054–0.172) and reduced the Brier score by 0.048 (95% CI, 0.024–0.070). The RBF-SVM fusion model achieved an AUC of 0.957; however, its incremental AUC over the corresponding baseline model was only 0.021 (95% CI, −0.003 to 0.050). Both sensitivity analyses yielded findings consistent in direction with the primary analysis, and inclusion of all seven quantitative parameters did not further improve model performance.

**Conclusion:**

LBI_SUVmax_ and LBI_SUVmean_ improved the discrimination between STB and PS when added to conventional clinical, laboratory, and imaging information and demonstrated consistent incremental value in the Elastic Net primary analysis and prespecified sensitivity analyses. Integrated quantitative SPECT/CT parameters may therefore provide useful complementary diagnostic information; however, their clinical applicability requires confirmation through multicenter external validation and prospective studies.

## 1 Introduction

Spinal tuberculosis (STB) and pyogenic spondylitis (PS) are two major forms of spinal infection, both of which can cause vertebral destruction, abscess formation, spinal deformity, and neurological impairment (Lee, 2014). Because they share several clinical manifestations, including fever, back pain, elevated inflammatory markers, and overlapping findings on conventional imaging, their early differentiation remains challenging (Jung et al., 2004; Chang et al., 2006). STB generally requires prolonged multidrug antituberculous therapy, whereas PS requires timely pathogen-directed antimicrobial treatment and, when necessary, appropriate source control (Liu et al., 2023). Accurate differentiation is therefore essential for selecting the appropriate treatment strategy, reducing diagnostic delay, and improving patient outcomes. The diagnosis of STB and PS currently relies on a combination of clinical assessment, laboratory inflammatory markers, microbiological testing, histopathological examination, and imaging evaluation. Inflammatory markers, including erythrocyte sedimentation rate (ESR), C-reactive protein (CRP), and procalcitonin (PCT), reflect infectious and inflammatory activity but have limited disease specificity (Piccolo et al., 2024; Yu et al., 2025). Although microbiological testing and tissue biopsy can provide definitive etiological evidence, their diagnostic utility may be constrained by suboptimal positivity rates, long turnaround times, sampling limitations, and procedural invasiveness (Kim, 2021; Haykır Solay et al., 2025). Recent studies have also demonstrated that metagenomic next-generation sequencing can provide additional information on pathogen composition and support the etiological diagnosis of vertebral osteomyelitis (Gao et al., 2024). MRI, CT, and radiography can demonstrate structural abnormalities associated with spinal infection; however, considerable overlap exists between the imaging features of STB and PS, limiting the discriminatory value of conventional imaging alone (Skaf et al., 2010; Dunn and Ben Husien, 2018). Consequently, reliance on a single source of clinical, laboratory, or imaging information may not provide sufficiently robust and accurate differentiation.

With advances in quantitative nuclear medicine imaging, ^99m^Tc-methylene diphosphonate (^99m^Tc-MDP) SPECT/CT has emerged as an objective and noninvasive approach for the quantitative assessment of spinal infections (Dickson et al., 2023). In addition to precise anatomical localization, quantitative SPECT/CT provides information on lesion-level bone metabolic activity and CT attenuation, allowing local bone metabolism to be quantified using parameters such as standardized uptake values (SUVs) (Mutuleanu et al., 2023). Previous studies have demonstrated the potential diagnostic value of quantitative SPECT/CT in the evaluation of bone infections and osteomyelitis and suggested that it may provide complementary information for clinical decision-making (Sontheimer et al., 2025). However, most previous studies have used an individual lesion or a single target vertebra as the unit of analysis, with limited consideration of the segmental distribution of spinal infection and the frequent involvement of adjacent vertebrae. Measurements derived from a single vertebra may not adequately reflect the peak metabolic intensity or overall lesion burden of the entire infected segment. Patient-level integrated quantitative parameters derived from adjacent involved vertebrae may therefore provide a more representative characterization of segmental disease activity and offer additional quantitative information for differentiating STB from PS.

Machine-learning methods can integrate demographic characteristics, laboratory markers, and multimodal imaging features within a unified predictive framework (Acosta et al., 2022; Hu et al., 2025; Liawrungrueang et al., 2025). Previous studies have developed models for differentiating STB from PS using laboratory markers or conventional imaging findings and have reported potentially useful diagnostic performance (Huang et al., 2024). However, the incremental diagnostic value of integrated quantitative SPECT/CT parameters beyond conventional clinical, laboratory, and imaging information has not been systematically evaluated. Moreover, the consistency of their incremental contribution across different modeling frameworks remains uncertain. Therefore, this study aimed to develop and internally validate multimodal models for differentiating STB from PS and to determine whether integrated quantitative SPECT/CT parameters provide additional diagnostic information beyond conventional predictors.

## 2 Materials and methods

### 2.1 Study population

This single-center retrospective study consecutively enrolled patients diagnosed with spinal tuberculosis or pyogenic spondylitis at the General Hospital of Ningxia Medical University between September 2024 and May 2026. All clinical data, laboratory measurements, conventional imaging findings, and SPECT/CT data included in the analysis were obtained before the definitive diagnosis was established. The study was approved by the Ethics Committee of the General Hospital of Ningxia Medical University and was conducted in accordance with the Declaration of Helsinki. Owing to the retrospective nature of the study, the requirement for written informed consent was waived by the ethics committee.

### 2.2 Inclusion and exclusion criteria

The inclusion criteria were as follows: (1) a definitive diagnosis of spinal tuberculosis (STB) or pyogenic spondylitis (PS) established by microbiological testing, molecular assays, including Xpert MTB/RIF or targeted next-generation sequencing (tNGS), or histopathological examination; (2) completion of ^99m^Tc-MDP SPECT/CT examination with image quality sufficient for quantitative analysis; and (3) complete clinical data, laboratory test results, and conventional imaging records.

The exclusion criteria were as follows: (1) spinal infection of another etiology or an indeterminate diagnosis; (2) mixed infection or infection involving multiple pathogens; or (3) concurrent spinal malignancy, autoimmune disease, or another condition that could substantially affect inflammatory markers or imaging assessment.

### 2.3 SPECT/CT scanning and image analysis

All patients underwent ^99m^Tc-MDP SPECT/CT according to the standard clinical imaging protocol. This retrospective study used archived images and examination records for subsequent image processing and quantitative analysis. Imaging was performed using a Symbia Intevo Bold SPECT/CT system (Siemens Healthineers, Germany). Each patient received an intravenous injection of 20–25 mCi of ^99m^Tc-MDP and was instructed to maintain adequate hydration and empty the bladder before image acquisition. Imaging was performed approximately 3 h after tracer administration. Patients were positioned supine and initially underwent whole-body bone scintigraphy, followed by regional SPECT/CT acquisition centered on the area of abnormal radiotracer uptake.

SPECT data were acquired using a 140-keV photopeak with a 20% energy window, a 256 × 256 matrix, an angular increment of 6°, and an acquisition time of 20 s per projection. CT was performed using a 16-slice helical acquisition with a tube voltage of 130 kV, a tube current of 160 mA, and a 512 × 512 matrix. The CT images were used for anatomical localization, attenuation correction, and quantitative measurement of CT attenuation in Hounsfield units (HU). Patient height and weight, pre- and post-injection syringe activity, activity measurement times, injection time, and scan time were recorded for SUV calculation and quantitative correction.

Image processing and quantitative measurements were performed using Esoft software on a Syngo workstation. Reconstructed SPECT images were coregistered and fused with the corresponding CT images. Areas of abnormal radiotracer uptake were identified on the fused images, and a volume of interest (VOI) was delineated for each involved vertebra. An initial VOI was generated using the software’s automated segmentation function and was subsequently reviewed and manually adjusted by nuclear medicine physicians to exclude adjacent normal bone, intervertebral discs, and non-target areas of increased uptake.

**Figure 1:**
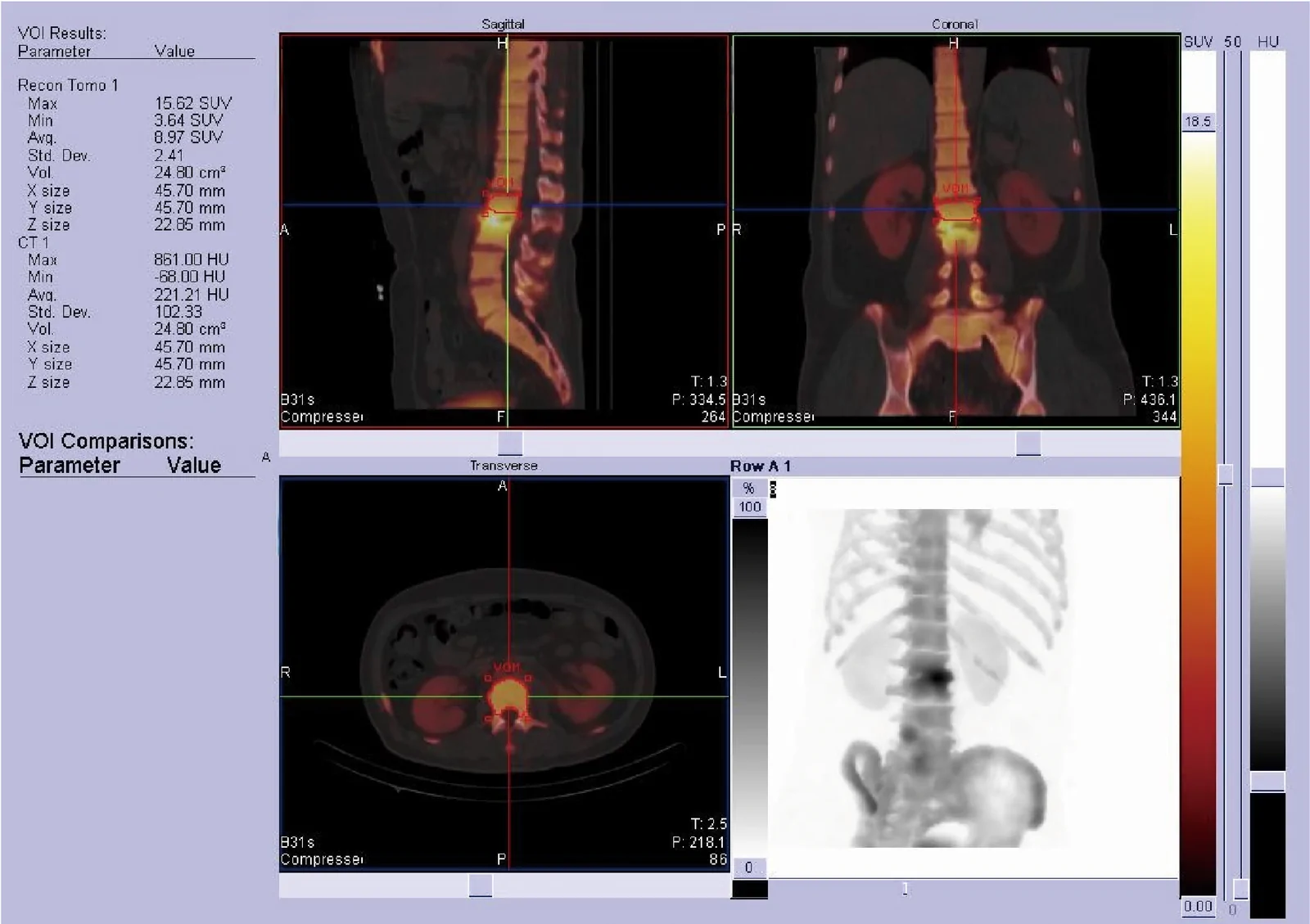
Quantitative SPECT/CT examination obtained in a patient with spinal tuberculosis involving the L2 and L3 vertebra. Fused SPECT/CT images in the sagittal, coronal, and axial planes, together with the corresponding planar bone scintigraphy image, demonstrate increased radiotracer uptake in the involved L2 and L3 vertebral lesion.

**Figure 2:**
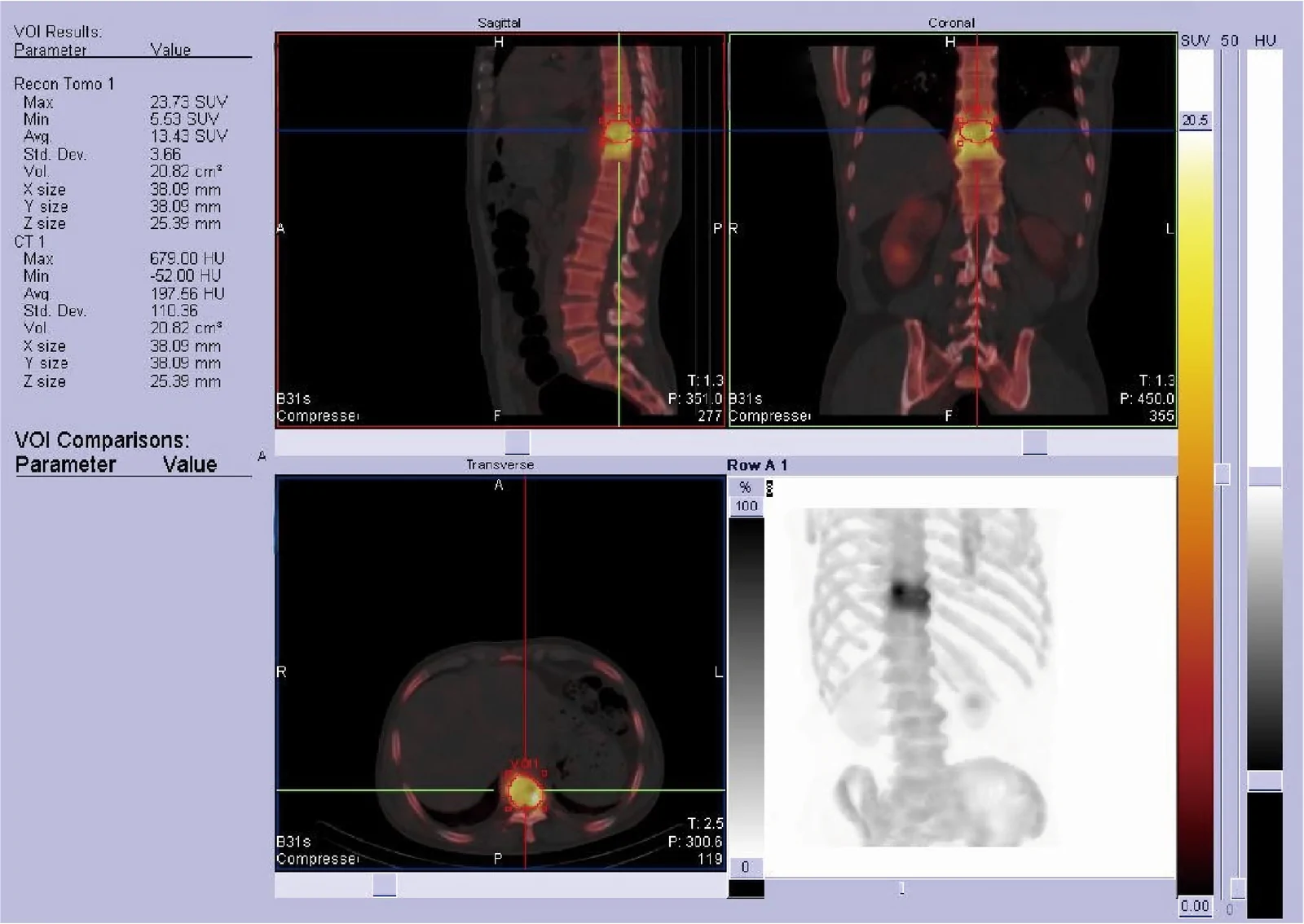
Quantitative SPECT/CT examination obtained in a patient with pyogenic spondylitis involving the T9 and T10 vertebra. Fused SPECT/CT images in the sagittal, coronal, and axial planes, together with the corresponding planar bone scintigraphy image, demonstrate increased radiotracer uptake in the involved T9 and T10 vertebral lesion.

The following parameters were extracted from the VOI of each involved vertebra: maximum, minimum, and mean standardized uptake values (SUVmax, SUVmin, and SUVmean); metabolic bone volume (MBV); total bone uptake (TBU); and maximum, minimum, and mean CT attenuation values (CT HUmax, CT HUmin, and CT HUmean), as illustrated in **Figures 1** and **2**. All images were independently evaluated by two experienced nuclear medicine physicians who were blinded to the patients’ final etiological diagnoses. The two readers independently performed lesion identification and VOI correction. Any disagreement was resolved by consensus.

### 2.4 Collection of clinical, laboratory, and conventional imaging features

Demographic characteristics, laboratory parameters, and conventional imaging features were collected for all patients. Demographic variables included sex, age, height, and weight. Height and weight were used exclusively for SUV calculation and quantitative correction and were not included as independent predictors in the machine-learning models.

For each patient, laboratory values obtained closest in time to the SPECT/CT examination were used. The original laboratory parameters included white blood cell count (WBC), neutrophil count (NEU), lymphocyte count (LYM), monocyte count (MONO), eosinophil count (EOS), basophil count (BASO), erythrocyte sedimentation rate (ESR), C-reactive protein (CRP), procalcitonin (PCT), red blood cell count (RBC), hemoglobin concentration (HGB), mean corpuscular hemoglobin concentration (MCHC), red cell distribution width–coefficient of variation (RDW-CV), platelet count (PLT), mean platelet volume (MPV), and platelet distribution width (PDW). Derived inflammatory indices were additionally calculated from the original blood cell counts, including the platelet-to-monocyte ratio (PMR), monocyte-to-lymphocyte ratio (MLR), neutrophil-to-lymphocyte ratio (NLR), platelet-to-lymphocyte ratio (PLR), platelet-to-neutrophil ratio (PNR), and systemic immune-inflammation index (SII), calculated as PLT × NEU/LYM. These derived indices were used only in a prespecified sensitivity analysis.

Conventional imaging variables were obtained from MRI, CT, and radiographic examinations. MRI features included spinal canal stenosis, paravertebral abscess, epidural abscess, and psoas abscess. CT features included vertebral destruction, marginal osteophyte formation, endplate sclerosis, spinal canal stenosis, paravertebral abscess, and epidural abscess. Radiographic features included intervertebral disc-space narrowing, endplate sclerosis, and osseous proliferation. All conventional imaging features were recorded as binary variables indicating the presence or absence of each finding.

### 2.5 Collection of quantitative SPECT/CT parameters and construction of integrated parameters

Quantitative SPECT/CT measurements comprised SUV-derived metabolic parameters and CT attenuation parameters. The SUV-derived parameters included SUVmax, SUVmin, SUVmean, TBU, and MBV, whereas the CT attenuation parameters included CT HUmax, CT HUmin, and CT HUmean. Because both STB and PS commonly exhibit segmental involvement with infection extending across adjacent vertebrae, two adjacent involved vertebrae within the same infected segment were defined as the patient-level unit of quantitative analysis. The two involved vertebrae were designated as V1 and V2, and their corresponding metabolic bone volumes were denoted as MBV1 and MBV2, respectively. Based on measurements from these two adjacent vertebrae, seven patient-level integrated quantitative SPECT/CT parameters were derived: LBI_SUVmax_, SUVmin_int_, LBI_SUVmean_, TBU_AM_, LBI_CT HUmax_, CT HUmin_int_, and LBI_CT HUmean_.

where LBI_SUVmax_ was defined as the MBV-weighted mean of SUVmax across the two involved vertebrae:


$$LBI_{suvmax}=\frac{SUVmax_{1}\times MBV_{1}+SUVmax_{2}\times MBV_{2}}{MBV_{1}+MBV_{2}}$$

The integrated minimum SUV, SUVmin_int_, was defined as the lower of the SUVmin values measured in the two involved vertebrae:


$$SUVmin_{int}=\min(SUVmin_{1},SUVmin_{2})$$

LBI_SUVmean_ was defined as the MBV-weighted mean of SUVmean across the two involved vertebrae:


$$LBI_{suvmean}=\frac{SUVmean_{1}\times MBV_{1}+SUVmean_{2}\times MBV_{2}}{MBV_{1}+MBV_{2}}$$

For each involved vertebra, TBU was calculated as the product of SUVmean and MBV:


$$TBU=SUV_{mean}\times MBV$$

The arithmetic mean of total bone uptake across the two involved vertebrae was denoted as TBU_AM_ and calculated as follows:


$$TBU_{AM}=\frac{TBU_{1}+TBU_{2}}{2}$$

LBI_CTHUmax_ was defined as the MBV-weighted mean of CT HUmax across the two involved vertebrae:


$$LBI_{CTHUmax}=\frac{CTHUmax_{1}\times MBV_{1}+CTHUmax_{2}\times MBV_{2}}{MBV_{1}+MBV_{2}}$$

The integrated minimum CT attenuation value, CT HUmin_int_, was defined as the lower of the CT HUmin values measured in the two involved vertebrae:


$$CTHUmin_{int}=min(CTHUmin_{1},CTHUmin_{2})$$

LBI_CTHUmean_ was defined as the MBV-weighted mean of CT HUmean across the two involved vertebrae:


$$LBI_{CTHUmean}=\frac{CTHUmean_{1}\times MBV_{1}+CTHUmean_{2}\times MBV_{2}}{MBV_{1}+MBV_{2}}$$

### 2.6 Data partitioning and feature selection

Model development and internal validation were performed using repeated stratified nested cross-validation. The outer loop consisted of stratified five-fold cross-validation repeated 20 times and was used to generate out-of-fold predictions and estimate model performance. Within each outer training set, stratified five-fold inner cross-validation was conducted for data preprocessing, hyperparameter optimization, and classification-threshold selection. The baseline, quantitative SPECT/CT-only, and fusion models were trained and evaluated using identical outer and inner data partitions to ensure paired comparisons across model specifications.

All preprocessing, feature handling, and model-selection procedures were confined to the relevant training folds to prevent information leakage. Within each training fold, missing values for continuous variables were imputed using the training-fold median, after which the variables were standardized using the corresponding training-fold mean and standard deviation. Missing categorical values were imputed using the training-fold mode, and categorical variables were encoded as indicator variables. The imputation, encoding, and scaling parameters estimated from the training data were then applied unchanged to the corresponding validation or held-out outer test fold.

After the optimal hyperparameters had been selected through inner cross-validation, each model was refitted using the complete outer training set and applied to the corresponding held-out outer test fold. Within each outer training set, the classification threshold was selected by maximizing the Youden index based on the inner cross-validation out-of-fold predictions. Because each patient appeared once in an outer test fold during each repetition, 20 out-of-fold predicted probabilities and 20 corresponding classification thresholds were obtained for each patient. These values were averaged at the patient level. Patients whose mean predicted probability was equal to or greater than their mean classification threshold were classified as PS; otherwise, they were classified as STB. The patient-level aggregated out-of-fold probabilities were used for the final evaluation of model performance.

Candidate predictors were prespecified on the basis of clinical relevance, data availability, and the study objectives, rather than selected according to univariate statistical significance. Features with zero variance, categorical predictors with fewer than five observations in any category, and variables that were completely redundant with other predictors were excluded before modeling. The primary analysis included the original laboratory measurements. Derived inflammatory indices were reserved for a prespecified sensitivity analysis, in which they replaced the corresponding original blood cell-count variables rather than being added alongside them. Elastic Net regularization was used to shrink coefficient estimates and perform variable selection among the candidate predictors.

### 2.7 Model development and performance evaluation

For all model analyses, PS was defined as the positive class and STB as the negative class. LBI_SUVmax_ and LBI_SUVmean_ were prespecified as the quantitative SPECT/CT features in the primary analysis, and three models were constructed: a baseline model, a quantitative SPECT/CT-only model, and a fusion model. The baseline model included age, sex, original laboratory parameters, and conventional imaging features. The quantitative SPECT/CT-only model included only LBI_SUVmax_ and LBI_SUVmean_. The fusion model incorporated these two parameters in addition to the baseline predictors. The incremental diagnostic value of the quantitative SPECT/CT parameters was assessed by comparing the performance of the fusion model with that of the baseline model.

Elastic Net logistic regression was used for the primary analysis. Candidate values for the inverse regularization parameter C were 0.01, 0.1, 0.5, 1.0, and 5.0, and candidate values for the L1 mixing parameter (l1_ratio) were 0, 0.25, 0.50, 0.75, and 1.0. In addition, a radial basis function support vector machine (RBF-SVM) was used for nonlinear robustness analysis, for which only the baseline and fusion models were constructed. For the RBF-SVM, candidate values for C were 0.1, 0.5, 1.0, 5.0, and 10.0, and candidate values for γ were scale, 0.001, 0.01, and 0.1. All optimal hyperparameters were selected through inner five-fold cross-validation. The primary performance measure was the area under the receiver operating characteristic curve (AUC), and secondary measures included the Brier score, accuracy, sensitivity, specificity, positive predictive value, negative predictive value, calibration intercept, and calibration slope.

Classification performance was calculated using patient-level aggregated out-of-fold predictions. Receiver operating characteristic curves and calibration curves were generated, and exploratory decision curve analysis was performed to assess the potential clinical net benefit of the models across a range of threshold probabilities.

The incremental value of quantitative SPECT/CT was evaluated using the difference in AUC and the reduction in Brier score between the fusion and baseline models. The AUC difference was defined as the AUC of the fusion model minus that of the baseline model, whereas the reduction in Brier score was defined as the Brier score of the baseline model minus that of the fusion model. Based on the patient-level aggregated out-of-fold predictions, 2, 000 paired bootstrap resamples were performed to estimate the 95% confidence intervals for both performance differences.

The feature stability of the Elastic Net fusion model was assessed by examining the frequency of nonzero coefficients, median coefficient values, and coefficient directions for each candidate feature across the 100 outer-loop fitted models. To evaluate the robustness of the primary findings, two prespecified sensitivity analyses based on Elastic Net were additionally conducted. In the first sensitivity analysis, the corresponding original blood cell counts were replaced with derived inflammatory indices, including PMR, MLR, NLR, PLR, PNR, and SII. In the second sensitivity analysis, all seven integrated quantitative SPECT/CT parameters were incorporated into the fusion model. Both sensitivity analyses followed the same data partitioning, preprocessing, inner-loop hyperparameter optimization, patient-level prediction aggregation, and performance-evaluation procedures as the primary analysis.

### 2.8 Statistical analysis

This study used Python software (version 3.12.4) for model development and statistical analysis. Continuous variables were summarized according to their distributions. Normally distributed continuous variables were expressed as the mean ± standard deviation and compared between groups using the independent-samples t-test. Non-normally distributed continuous variables were expressed as the median and interquartile range and compared using the Mann–Whitney U test. Categorical variables were presented as counts and percentages and compared using the χ² test or Fisher’s exact test, as appropriate. The 95% confidence intervals for model performance measures, as well as for the differences in AUC and reductions in Brier score between the fusion and baseline models, were estimated using patient-level bootstrap resampling. All statistical tests were two-sided, and a *P* value < 0.05 was considered statistically significant.

## 3 Results

### 3.1 Patient baseline characteristics

A total of 165 patients with spinal infection were included, comprising 96 patients with STB and 69 with PS. Clinical characteristics, laboratory measurements, conventional imaging findings, and quantitative SPECT/CT parameters were available for all patients. No significant differences in age or sex were observed between the two groups (both *P* > 0.05). Laboratory analysis showed that NEU, ESR, CRP, PCT, MLR, NLR, PLR, and SII were significantly higher in the PS group than in the STB group, whereas LYM was significantly higher in the STB group than in the PS group (all *P* < 0.01). No significant between-group differences were observed in the remaining laboratory parameters (**Table 1**).

**Table 1:** Demographic and laboratory characteristics of patients with STB and PS.

Variable	STB group (n = 96)	PS group (n = 69)	*P* value
Demographic characteristics			
Age, years	57.50 (50.25, 67.25)	55.00 (51.00, 63.50)	0.37
Sex, male/female, n	43/53	27/42	0.52
Weight, kg	59.00 (52.00, 65.00)	59.00 (53.50, 67.00)	0.44
Height, m	1.65 (1.61, 1.74)	1.66 (1.60, 1.72)	0.89
BMI, kg/m^2^	20.75 (18.20, 24.07)	21.48 (19.30, 24.84)	0.34
Laboratory indicators			
WBC, ×10^9^/L	10.43 (9.70, 11.17)	10.75 (9.59, 11.83)	0.18
NEU, ×10^9^/L	7.82 (6.78, 8.74)	8.30 (7.07, 10.34)	0.01
LYM, ×10^9^/L	1.44 (1.04, 1.75)	1.14 (0.65, 1.52)	0.01
MONO, ×10^9^/L	0.60 (0.46, 0.72)	0.62 (0.49, 0.77)	0.47
EOS, ×10^9^/L	0.52 (0.34, 0.77)	0.59 (0.30, 1.24)	0.30
BASO, ×10^9^/L	0.06 (0.05, 0.07)	0.07 (0.02, 0.13)	0.23
ESR, mm/h	44.00 (37.00, 49.75)	48.00 (42.00, 54.00)	0.01
CRP, mg/L	22.30 (20.10, 24.35)	27.70 (24.90, 31.65)	0.01
PCT, ng/mL	0.22 (0.14, 0.30)	0.33 (0.22, 0.56)	0.01
RBC, ×10^12^/L	4.72 (4.37, 5.06)	4.82 (4.40, 5.27)	0.24
HGB, g/L	120.62 (111.82, 131.89)	125.88 (111.57, 133.69)	0.55
MCHC, g/L	343.34 (337.68, 348.86)	345.46 (339.88, 353.18)	0.28
RDW-CV, %	25.00 (23.00, 29.00)	25.00 (22.00, 28.00)	0.88
PLT, ×10^9^/L	342.10 (294.56, 385.13)	343.11 (317.81, 381.71)	0.21
MPV, fL	7.16 (5.01, 8.26)	6.95 (5.27, 9.02)	0.78
PDW, %	15.00 (13.00, 17.00)	15.00 (12.00, 16.00)	0.26
Derived inflammatory indices			
PMR	596.05 (433.02, 755.37)	519.95 (423.68, 742.64)	0.81
MLR	0.43 (0.28, 0.61)	0.57 (0.43, 0.93)	0.01
NLR	5.56 (4.43, 7.62)	8.15 (4.86, 13.27)	0.01
PLR	232.21 (185.44, 322.37)	323.68 (220.16, 541.09)	0.01
PNR	45.65 (35.42, 52.85)	42.63 (34.08, 48.20)	0.29
SII, ×10^9^/L	1863.35 (1338.12, 2504.88)	3137.90 (1696.03, 4987.31)	0.01

Data are presented as median (interquartile range) unless otherwise indicated. Sex is presented as male/female counts. PMR, MLR, NLR, PLR, and PNR are dimensionless ratios. STB, spinal tuberculosis; PS, pyogenic spondylitis; BMI, body mass index; ESR, erythrocyte sedimentation rate; CRP, C-reactive protein; PCT, procalcitonin; RDW-CV, red cell distribution width–coefficient of variation; SII, systemic immune-inflammation index.

### 3.2 Comparison of quantitative SPECT/CT parameters

Quantitative comparison of the SPECT/CT parameters showed that LBI_SUVmax_, SUVmin_int_, LBI_SUVmean_, TBU_AM_, LBI_CT HUmax_, CTHUmin_int_, and LBI_CT HUmean_ were all significantly higher in the PS group than in the STB group (all P < 0.01) (**Table 2**). Specifically, the median LBI_SUVmax_ values were 16.64 (14.30–18.31) in the STB group and 20.27 (18.02–21.89) in the PS group, whereas the corresponding median LBI_SUVmean_ values were 8.62 (7.71–10.02) and 11.85 (9.88–13.73), respectively. Both parameters differed significantly between the two groups (both P < 0.01).

**Table 2:** Quantitative SPECT/CT integrated parameters and conventional imaging features of patients with STB and PS.

Variable	STB group (n = 96)	PS group (n = 69)	*P* value
Quantitative SPECT/CT integrated parameters			
LBI_SUVmax_	16.64 (14.30, 18.31)	20.27 (18.02, 21.89)	0.01
SUVmin_int_	3.33 (3.02, 4.02)	3.68 (3.37, 4.13)	0.01
LBI_SUVmean_	8.62 (7.71, 10.02)	11.85 (9.88, 13.73)	0.01
TBU_AM_	283.01 (221.98, 363.92)	402.56 (300.43, 521.65)	0.01
LBI_CT HUmax_	685.50 (584.50, 816.00)	877.00 (741.00, 966.00)	0.01
CT HUmin_int_	-45.50 (-58.00, -36.00)	-39.00 (-44.00, -35.00)	0.01
LBI_CT HUmean_	254.88 (209.20, 301.18)	292.33 (245.94, 334.64)	0.01
MRI features			
Spinal canal stenosis, n (%)	51/96 (53.1)	34/69 (49.3)	0.63
Paravertebral abscess, n (%)	69/96 (71.9)	35/69 (50.7)	0.01
Epidural abscess, n (%)	49/96 (51.0)	39/69 (56.5)	0.49
Psoas abscess, n (%)	29/96 (30.2)	30/69 (43.5)	0.08
CT features			
Vertebral destruction, n (%)	44/96 (45.8)	29/69 (42.0)	0.63
Marginal osteophytes, n (%)	28/96 (29.2)	20/69 (29.0)	0.98
Endplate sclerosis, n (%)	32/96 (33.3)	20/69 (29.0)	0.55
Spinal canal stenosis, n (%)	34/96 (35.4)	30/69 (43.5)	0.29
Paravertebral abscess, n (%)	44/96 (45.8)	25/69 (36.2)	0.22
Epidural abscess, n (%)	34/96 (35.4)	29/69 (42.0)	0.39
X-ray features			
Disc space narrowing, n (%)	37/96 (38.5)	20/69 (29.0)	0.20
Endplate sclerosis, n (%)	24/96 (25.0)	22/69 (31.9)	0.33
Hyperostosis, n(%)	37/96 (38.5)	25/69 (36.2)	0.76

### 3.3 Elastic net primary analysis

**Figure 3:**
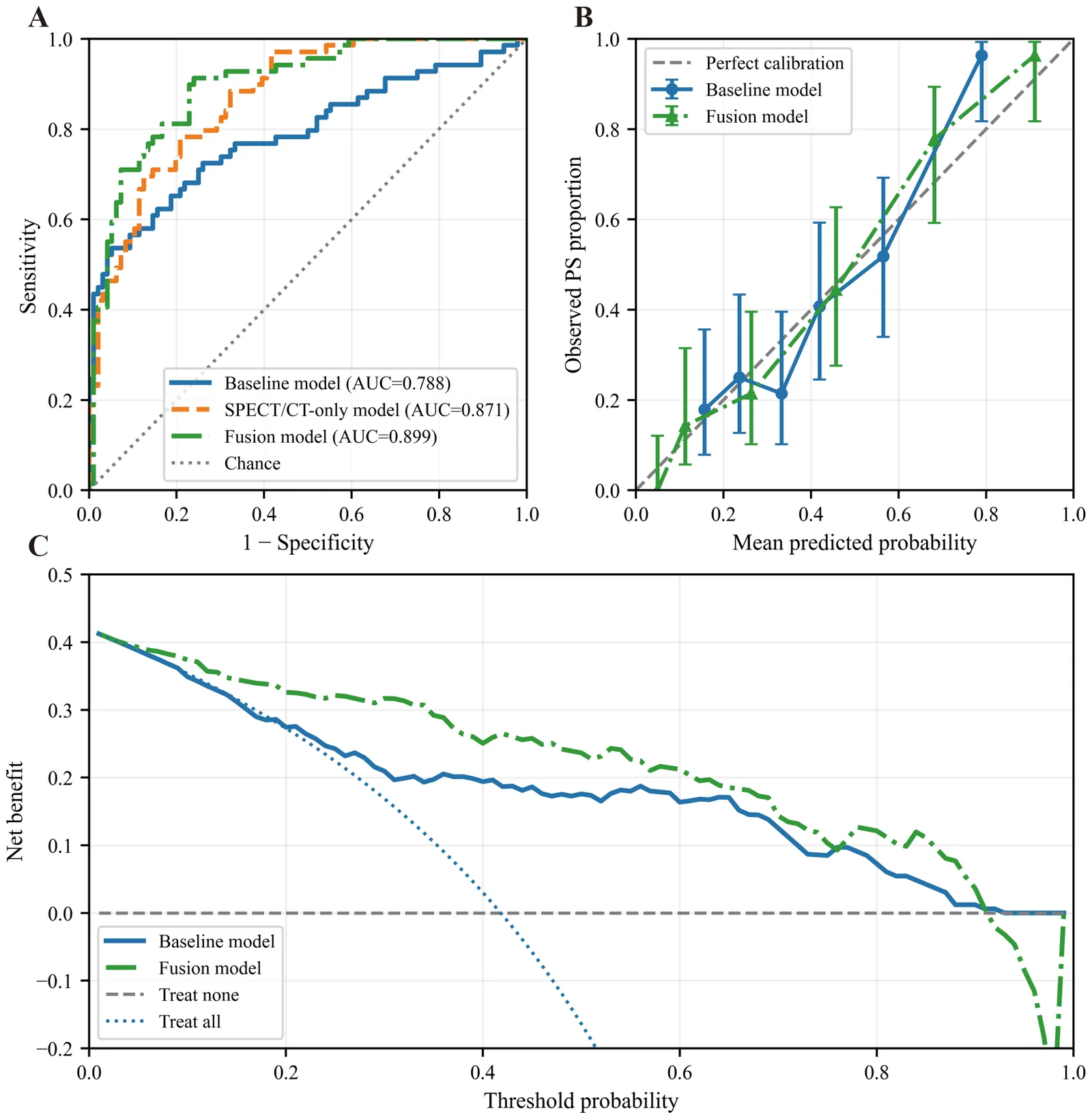
Discrimination, calibration, and decision-curve analysis of the primary Elastic Net models based on patient-level aggregated out-of-fold predictions. **(A)** Receiver operating characteristic curves for the baseline, SPECT/CT-only, and fusion models. **(B)** Calibration curves for the baseline and fusion models. Error bars represent Wilson 95% confidence intervals for the observed proportions of pyogenic spondylitis. **(C)** Exploratory decision-curve analysis comparing the baseline and fusion models across a range of threshold probabilities.

In the Elastic Net primary analysis, the AUCs of the baseline, quantitative SPECT/CT-only, and fusion models were 0.788 (95% CI, 0.711–0.863), 0.871 (95% CI, 0.815–0.920), and 0.899 (95% CI, 0.847–0.944), respectively. The fusion model achieved the highest AUC among the three models (**Table 3**; **Figure 3A**). The corresponding Brier scores were 0.176, 0.155, and 0.128, respectively. The fusion model achieved an accuracy of 80.6%, sensitivity of 79.7%, specificity of 81.2%, positive predictive value of 75.3%, and negative predictive value of 84.8%. Compared with the baseline model, the fusion model increased accuracy from 74.5% to 80.6%, sensitivity from 58.0% to 79.7%, and negative predictive value from 74.1% to 84.8%, whereas specificity decreased slightly from 86.5% to 81.2%. Based on the patient-level aggregated out-of-fold predictions, the baseline model correctly identified 40 patients with PS but missed 29 cases. The fusion model correctly identified 55 patients with PS and missed 14 cases, thereby correctly identifying 15 additional PS cases compared with the baseline model. Meanwhile, the number of patients with STB misclassified as PS increased from 13 to 18. Paired bootstrap analysis showed that, compared with the baseline model, the fusion model increased the AUC by 0.110 (95% CI, 0.054–0.172) and reduced the Brier score by 0.048 (95% CI, 0.024–0.070). The 95% confidence intervals for both paired performance differences excluded zero (**Table 4**).

**Table 3:** Performance of the machine-learning models based on patient-level aggregated out-of-fold predictions.

Algorithm	Model	AUC (95% CI)	Brier score (95% CI)	Accuracy (%)	Sensitivity (%)	Specificity (%)	PPV (%)	NPV (%)	Calibration intercept/slope
Elastic Net	Baseline	0.788 (0.711-0.863)	0.176 (0.149-0.205)	74.5	58.0	86.5	75.5	74.1	0.143/1.330
	SPECT-only	0.871 (0.815-0.920)	0.155 (0.133-0.177)	75.2	76.8	74.0	67.9	81.6	0.220/1.889
	Fusion	0.899 (0.847-0.944)	0.128 (0.100-0.159)	80.6	79.7	81.2	75.3	84.8	0.137/1.187
RBF-SVM	Baseline	0.937 (0.898-0.970)	0.097 (0.071-0.123)	87.3	84.1	89.6	85.3	88.7	−0.122/1.252
	Fusion	0.957 (0.927-0.981)	0.084 (0.062-0.111)	86.7	84.1	88.5	84.1	88.5	−0.370/1.370

PS was defined as the positive class. PPV, positive predictive value; NPV, negative predictive value; CI, confidence interval. Classification metrics were calculated using mean predicted probability ≥ mean fold-specific threshold.

**Table 4:** Incremental value of quantitative SPECT/CT parameters and sensitivity analyses.

Analysis	Algorithm	Baseline AUC	Fusion AUC	ΔAUC (95% CI)	Baseline Brier	Fusion Brier	Brier improvement (95% CI)
Primary analysis	Elastic Net	0.788	0.899	0.110 (0.054–0.172)	0.176	0.128	0.048 (0.024–0.070)
Nonlinear robustness analysis	RBF-SVM	0.937	0.957	0.021 (−0.003–0.050)	0.097	0.084	0.012 (−0.005–0.030)
Sensitivity analysis 1: derived inflammatory indices	Elastic Net	0.810	0.910	0.101 (0.048–0.159)	0.164	0.122	0.041 (0.022–0.062)
Sensitivity analysis 2: all 7 SPECT/CT parameters	Elastic Net	0.788	0.892	0.104 (0.047–0.165)	0.176	0.132	0.044 (0.021–0.067)

The baseline model included age, sex, laboratory variables, and conventional imaging features. ΔAUC was calculated as AUC_fusion − AUC_baseline, and Brier score improvement as Brier_baseline − Brier_fusion. Positive values favor the fusion model. Confidence intervals were derived from 2, 000 patient-level paired bootstrap resamples.

### 3.4 RBF-SVM robustness analysis

**Figure 4:**
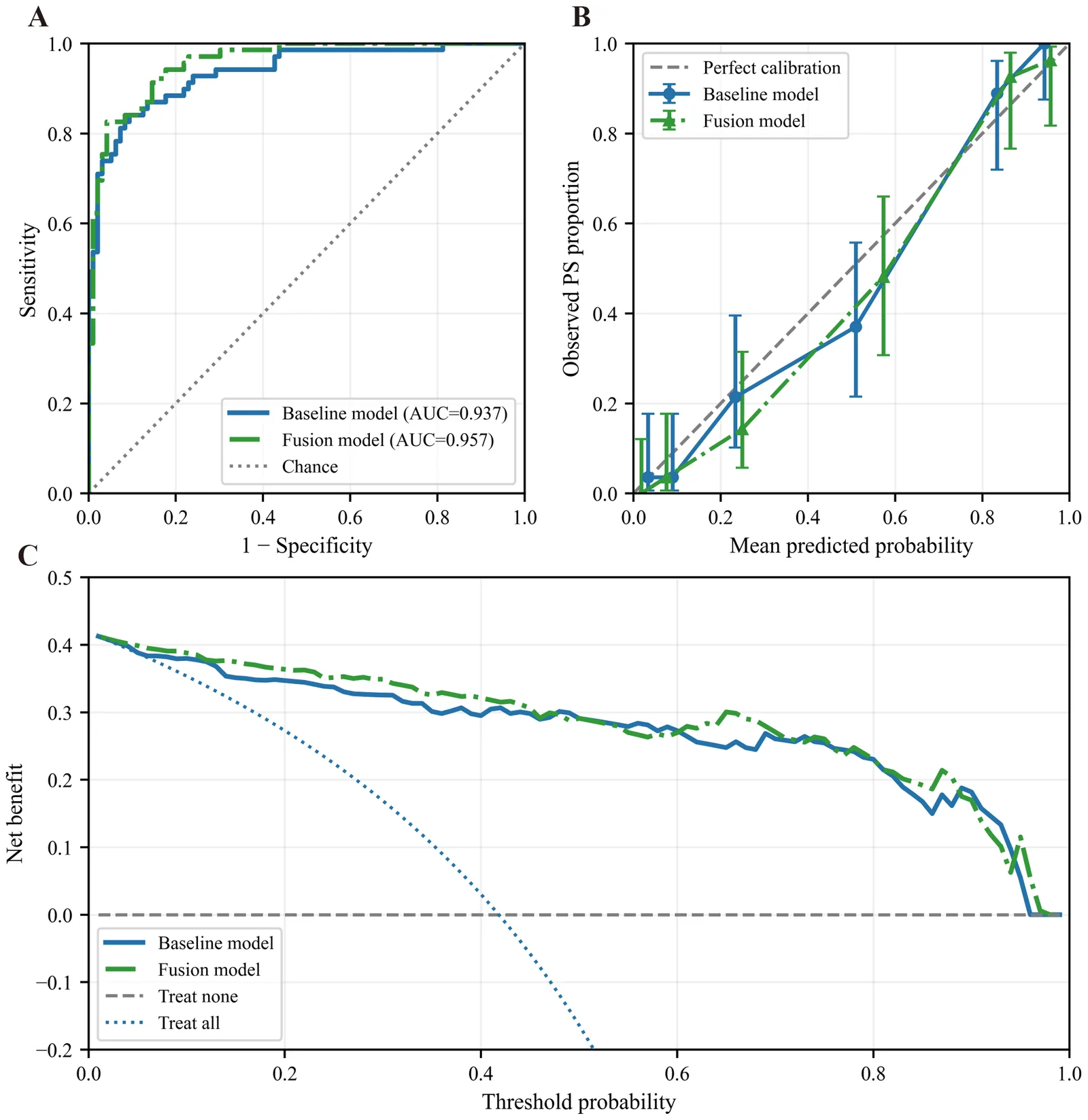
Discrimination, calibration, and decision-curve analysis of the RBF-SVM robustness models based on patient-level aggregated out-of-fold predictions. **(A)** Receiver operating characteristic curves for the baseline and fusion models. **(B)** Calibration curves for the baseline and fusion models. Error bars represent Wilson 95% confidence intervals for the observed proportions of pyogenic spondylitis. **(C)** Exploratory decision-curve analysis comparing the baseline and fusion models across a range of threshold probabilities.

In the RBF-SVM analysis, the AUCs of the baseline and fusion models were 0.937 (95% CI, 0.898–0.970) and 0.957 (95% CI, 0.927–0.981), respectively (**Table 3**; **Figure 4A**). The corresponding Brier scores were 0.097 and 0.084. Both models achieved a sensitivity of 84.1%, whereas the specificities of the baseline and fusion models were 89.6% and 88.5%, and their accuracies were 87.3% and 86.7%, respectively. Paired bootstrap analysis showed that, compared with the baseline model, the fusion model increased the AUC by 0.021 (95% CI, −0.003 to 0.050) and reduced the Brier score by 0.012 (95% CI, −0.005 to 0.030). The 95% confidence intervals for both paired performance differences included zero (**Table 4**).

### 3.5 Model calibration and decision curve analysis

The calibration intercepts of the Elastic Net baseline, quantitative SPECT/CT-only, and fusion models were 0.143, 0.220, and 0.137, respectively, and the corresponding calibration slopes were 1.330, 1.889, and 1.187 (**Table 3**). Among the three models, the fusion model had a calibration slope closest to the ideal value of 1. **Figure 3B** presents the calibration curves of the Elastic Net baseline and fusion models. For the RBF-SVM analysis, the calibration intercepts of the baseline and fusion models were −0.122 and −0.370, respectively, and the corresponding calibration slopes were 1.252 and 1.370 (**Table 3**; **Figure 4B**). Exploratory decision curve analysis showed that the Elastic Net fusion model provided a higher net benefit than the baseline model across most of the evaluated threshold-probability range (**Figure 3C**). The decision curves of the RBF-SVM baseline and fusion models were generally similar, with only minor differences across certain threshold-probability ranges (**Figure 4C**).

### 3.6 Sensitivity analyses

When derived inflammatory indices, including PMR, MLR, NLR, PLR, PNR, and SII, were substituted for the corresponding original blood cell-count variables, the AUCs of the Elastic Net baseline and fusion models were 0.810 and 0.910, respectively, and their Brier scores were 0.164 and 0.122. Compared with the baseline model, the fusion model increased the AUC by 0.101 (95% CI, 0.048–0.159) and reduced the Brier score by 0.041 (95% CI, 0.022–0.062) (**Table 4**). When all seven integrated quantitative SPECT/CT parameters were included in the fusion model, the AUCs of the baseline and fusion models were 0.788 and 0.892, respectively, and their Brier scores were 0.176 and 0.132. Compared with the baseline model, the fusion model increased the AUC by 0.104 (95% CI, 0.047–0.165) and reduced the Brier score by 0.044 (95% CI, 0.021–0.067) (**Table 4**). In both sensitivity analyses, the fusion model achieved a higher AUC and a lower Brier score than the corresponding baseline model, and the 95% confidence intervals for the paired performance differences excluded zero. The prespecified two-parameter fusion model achieved an AUC of 0.899 and a Brier score of 0.128. After all seven integrated quantitative SPECT/CT parameters were incorporated, the fusion model achieved an AUC of 0.892 and a Brier score of 0.132, indicating no further improvement in either performance measure.

### 3.7 Elastic net feature stability analysis

Across the 100 outer-loop Elastic Net fusion models, LBI_SUVmax_ and LBI_SUVmean_ retained nonzero coefficients in 100% of the fitted models. Their median coefficients were 0.829 and 0.842, respectively, and both were positive (**Table 5**). LYM, BASO, CRP, paravertebral abscess on MRI, NEU, and PLT retained nonzero coefficients in 98%, 96%, 95%, 94%, 92%, and 91% of the models, respectively. BASO, CRP, NEU, and PLT predominantly had positive coefficients, whereas LYM and paravertebral abscess on MRI predominantly had negative coefficients. MONO retained a nonzero coefficient in 83% of the models and generally had a positive coefficient. Because PS was defined as the positive class, positive coefficients were associated with higher predicted probabilities of PS, whereas negative coefficients were associated with lower predicted probabilities of PS.

**Table 5:** Coefficient stability in the primary elastic net fusion model across repeated outer-fold analyses.

Feature	Non-zero coefficient frequency (%)	Median coefficient	IQR	Coefficient direction
LBI_SUVmax_	100	0.829	[0.633, 1.119]	Positive
LBI_SUVmean_	100	0.842	[0.608, 1.059]	Positive
LYM	98	-0.406	[-0.538, -0.327]	Negative
BASO	96	0.365	[0.249, 0.545]	Positive
CRP	95	0.252	[0.189, 0.376]	Positive
MRI paravertebral abscess	94	-1.292	[-1.573, -0.372]	Negative
NEU	92	0.157	[0.085, 0.216]	Positive
PLT	91	0.171	[0.088, 0.244]	Positive
MONO	83	0.155	[0.040, 0.265]	Positive
MPV	83	-0.129	[-0.220, -0.038]	Negative

The table presents the 10 predictors with the highest non-zero coefficient frequencies across 100 outer-fold Elastic Net fusion models. Continuous predictors were standardized within each training fold, whereas categorical predictors were dummy-coded. Positive coefficients indicate a higher predicted probability of PS, and negative coefficients indicate a lower predicted probability of PS. Coefficients represent multivariable predictive associations rather than independent or causal effects. IQR, interquartile range; PS, pyogenic spondylitis.

## 4 Discussion

This study developed and internally validated multimodal machine-learning models integrating clinical characteristics, laboratory parameters, conventional imaging features, and quantitative SPECT/CT parameters in 165 patients with STB or PS confirmed by microbiological, molecular, or histopathological examinations. The primary findings showed that the quantitative SPECT/CT-only model incorporating LBI_SUVmax_ and LBI_SUVmean_ achieved good discrimination. In the Elastic Net primary analysis, adding these two parameters to the baseline model improved both discrimination and overall probabilistic accuracy, and the two prespecified sensitivity analyses yielded directionally consistent results. In contrast, although the RBF-SVM fusion model achieved the highest absolute AUC, its incremental improvement over the already high-performing baseline model was limited and uncertain. These findings suggest that the incremental contribution of quantitative SPECT/CT parameters may vary according to the modeling algorithm and the performance of the corresponding baseline model.

Previous quantitative SPECT/CT studies have generally used individual lesions or single target vertebrae as the unit of analysis, focusing primarily on local radiotracer uptake or differences among lesion types, with relatively limited consideration of the segmental distribution of spinal infection and the involvement of adjacent vertebrae (Qi et al., 2021). PS is typically associated with acute pyogenic inflammation and may be accompanied by marked local inflammatory activity, increased perfusion, and reactive osteogenesis (Placide and Reznicek, 2025). By contrast, STB is generally characterized by chronic granulomatous inflammation, caseous necrosis, and more heterogeneous patterns of osseous destruction (Rajasekaran et al., 2018). Because both conditions may involve adjacent vertebrae within the same infected segment, SUV or CT attenuation measurements obtained from a single vertebra may not adequately represent the overall metabolic activity and lesion burden of the entire affected segment (Ling-Shan et al., 2024). LBI_SUVmax_ and LBI_SUVmean_, derived from two adjacent involved vertebrae, were designed to reflect the peak metabolic intensity and average metabolic burden across the infected segment, respectively. These parameters may therefore capture pathophysiological differences that are not fully represented by conventional structural imaging. Both LBI_SUVmax_ and LBI_SUVmean_ retained positive nonzero coefficients in all 100 outer-loop Elastic Net fusion models, demonstrating consistent feature retention and coefficient direction across repeated model fitting.

The study findings not only highlight the complementary value of multimodal information but also demonstrate that the incremental contribution of quantitative SPECT/CT parameters differed across modeling algorithms. In the Elastic Net analysis, the baseline model achieved an AUC of 0.788, indicating that clinical, laboratory, and conventional imaging features provided moderate discrimination. After LBI_SUVmax_ and LBI_SUVmean_ were added, the AUC of the fusion model increased to 0.899, the Brier score decreased, and the calibration slope moved closer to the ideal value of 1. These findings suggest that the quantitative imaging parameters improved both discrimination between STB and PS and the overall accuracy of predicted probabilities. By contrast, the RBF-SVM baseline model already achieved an AUC of 0.937, which increased to 0.957 after the two SPECT/CT parameters were added; however, the 95% confidence interval for the paired AUC difference included zero, leaving the incremental benefit uncertain. Through its kernel function, RBF-SVM can capture nonlinear relationships and complex interactions among predictors (Noble, 2006). It is therefore possible that the clinical, laboratory, and conventional imaging features had already been more extensively exploited within this modeling framework, thereby limiting the additional contribution of the quantitative SPECT/CT parameters. Taken together, these findings suggest that the incremental value of quantitative SPECT/CT parameters may depend jointly on the performance of the baseline model and the characteristics of the modeling algorithm, which may explain the different magnitudes of improvement observed in the Elastic Net and RBF-SVM analyses.

Feature stability analysis further suggested that the incremental value of the quantitative SPECT/CT parameters did not diminish the relevance of conventional diagnostic information, as inflammatory markers and routine imaging features continued to provide complementary information within the fusion model. CRP, NEU, and PLT predominantly retained positive coefficients across the repeated outer-loop models, consistent with the more pronounced systemic inflammatory response commonly observed in PS. In contrast, LYM generally showed a negative coefficient, which may reflect differences in host immune-response patterns between STB and PS. Previous studies have also suggested that peripheral blood inflammatory indices, including NLR and MLR, may contribute to differentiating STB from PS and assessing disease severity (Chen et al., 2022; Liu et al., 2022). Among the conventional imaging features, paravertebral abscess on MRI predominantly retained a negative coefficient, indicating that this finding tended to support the prediction of STB in the multivariable model. This result is consistent with the characteristic imaging appearance of paravertebral abscesses in STB, which often have a larger extent, relatively well-defined margins, and a chronic disease course (Park et al., 2011). It should be emphasized that Elastic Net coefficients represent predictive associations in a multivariable context rather than independent effects or causal relationships. Therefore, variables with limited mechanistic support, such as BASO, should be interpreted cautiously.

Two prespecified sensitivity analyses further supported the robustness of the primary findings. After the corresponding original blood cell-count variables were replaced with derived inflammatory indices, the addition of LBI_SUVmax_ and LBI_SUVmean_ continued to improve model performance. When all seven integrated quantitative SPECT/CT parameters were included in the fusion model, performance also improved compared with the baseline model; however, neither the AUC nor the Brier score exceeded that of the prespecified two-parameter fusion model. These findings suggest that, in a study with a limited sample size, simply increasing the number of quantitative predictors may not provide additional predictive benefit and may instead increase model complexity because of greater intercorrelation among variables and less stable coefficient estimation. Compared with the seven-parameter model, the addition of LBI_SUVmax_ and LBI_SUVmean_ alone may therefore provide a more favorable balance among predictive performance, interpretability, and reproducibility.

The study findings also suggest that integrated quantitative SPECT/CT parameters may have potential clinical value. The improvement observed with the Elastic Net fusion model was primarily reflected in a reduction in false-negative classifications of PS, which may be clinically relevant because STB and PS require different treatment strategies. When microbiological evidence is inconclusive or conventional imaging findings overlap, LBI_SUVmax_ and LBI_SUVmean_ may provide complementary information to existing clinical, laboratory, and imaging data and thereby support further diagnostic assessment and clinical decision-making. Exploratory decision curve analysis showed that the fusion model provided a greater net benefit than the baseline model across most of the evaluated threshold-probability range, suggesting that it may have potential to improve risk stratification and support more informed clinical decisions.

This study has several limitations. First, it was a single-center retrospective study with a relatively small sample size, which may have introduced selection bias related to patient inclusion and disease distribution. Although rigorous internal validation was performed using repeated stratified nested cross-validation, internal validation cannot substitute for independent external validation. Therefore, the generalizability of the models to other patient populations and healthcare settings remains uncertain. Second, quantitative SPECT/CT measurements may be affected by scanner type, acquisition and reconstruction protocols, quantitative calibration procedures, and volume-of-interest delineation methods. Further standardization and reproducibility studies are therefore required before these parameters can be applied across multiple centers. Third, conventional imaging features were recorded as binary variables, which may not fully capture lesion extent, morphology, or severity. Finally, prospective clinical validation and decision-impact assessment were not performed. The actual clinical utility of the models therefore remains to be confirmed in large-scale multicenter and prospective studies.

The findings indicate that adding LBI_SUVmax_ and LBI_SUVmean_ to clinical characteristics, laboratory measurements, and conventional imaging information improved discrimination between STB and PS. This incremental value was consistently observed in the primary Elastic Net analysis and the prespecified sensitivity analyses, although the relative improvement was more limited and uncertain in the RBF-SVM analysis. Integrated quantitative SPECT/CT parameters may therefore provide useful complementary information to conventional diagnostic data. However, the external generalizability and clinical applicability of the models require further confirmation through multicenter external validation and prospective studies.

## Data Availability

The datasets generated and/or analyzed during the current study are not readily available because they contain patient information and are subject to institutional ethical and privacy restrictions. Requests to access the datasets should be directed to Ningkui Niu (niuningkui6743242@163.com).
